# Feasibility of creating a daily adaptive plan using automatic DIR-created target and OARs contours in patients with prostate cancer magnetic-resonance-guided adaptive radiotherapy

**DOI:** 10.1093/jrr/rrae088

**Published:** 2024-11-11

**Authors:** Makoto Saito, Kota Abe, Masato Tsuneda, Yukio Fujita, Yukinao Abe, Tsumugi Nishimura, Asuka Kodate, Aki Kanazawa, Rintaro Harada, Miho Watanabe, Takashi Uno

**Affiliations:** Department of Radiology, Chiba University Hospital, 1-8-1 Inohana, Chuo-ku, Chiba 260-8677, Japan; Department of Radiation Oncology, MR Linac ART Division, Graduate School of Medicine, Chiba University, 1-8-1 Inohana, Chuo-ku, Chiba 260-8670, Japan; Department of Radiation Oncology, MR Linac ART Division, Graduate School of Medicine, Chiba University, 1-8-1 Inohana, Chuo-ku, Chiba 260-8670, Japan; Department of Radiation Oncology, MR Linac ART Division, Graduate School of Medicine, Chiba University, 1-8-1 Inohana, Chuo-ku, Chiba 260-8670, Japan; Department of Radiation Sciences, Komazawa University, Setagaya, Tokyo, 259-1193, Japan; Department of Radiology, Chiba University Hospital, 1-8-1 Inohana, Chuo-ku, Chiba 260-8677, Japan; Department of Radiology, Chiba University Hospital, 1-8-1 Inohana, Chuo-ku, Chiba 260-8677, Japan; Department of Radiology, Chiba University Hospital, 1-8-1 Inohana, Chuo-ku, Chiba 260-8677, Japan; Department of Radiology, Chiba University Hospital, 1-8-1 Inohana, Chuo-ku, Chiba 260-8677, Japan; Department of Radiology, Chiba University Hospital, 1-8-1 Inohana, Chuo-ku, Chiba 260-8677, Japan; Diagnostic Radiology and Radiation Oncology, Graduate School of Medicine, Chiba University, 1-8-1 Inohana, Chuo-ku, Chiba 260-8670, Japan; Diagnostic Radiology and Radiation Oncology, Graduate School of Medicine, Chiba University, 1-8-1 Inohana, Chuo-ku, Chiba 260-8670, Japan

**Keywords:** MR-Linac, prostate cancer, MR-guided online adaptive radiotherapy, deformable image registration, stereotactic body radiotherapy (SBRT)

## Abstract

The purpose of this study was to evaluate the feasibility of treatment plans for prostate cancer with magnetic resonance (MR)-guided online adaptive radiotherapy, which are generated using deformable image registration (DIR)-created contours of the targets and organs. Totally, 150 fractions from 30 prostate cancer patients implanted with a hydrogel spacer and treated with the MR-Linac were studied. Reference treatment plans that satisfied all institutional dose constraints were initially created on planning MRI. The adaptive treatment plans were created on daily MRI based on the reference plan using the DIR-created contours, ensuring all dose constraints were met. Subsequently, a clinician manually created reference contours for each daily MRI. Finally, the dose volume histogram indices of the plan generated with DIR-created contours were re-evaluated with clinician created contours. The evaluated contours included the bladder wall, rectum wall, sigmoid, small bowel and planning target volume (PTV) for dose prescription. The PTV for dose prescription met the dose constraints in all fractions. The bladder and rectum walls met the dose constraint of maximum dose (D_0.03 cc_) in all fractions. Five patients failed to meet the sigmoid and small bowel dose constraints, with the largest deviation being 13.3% exceedance at D_2 cc_ in the small bowel added 3 mm margin. This study suggests that most treatment plans created without modifying the DIR-created contours are clinically viable. However, dislodgements of the small bowel and sigmoid may exceed the extent of DIR propagation from the reference plan contours, and it is recommended that these contours be verified.

## INTRODUCTION

Radiotherapy for prostate cancer can now be performed in five fractions or less, a significant reduction from the 40 fractions previously required. This advancement is largely due to developments in intensity-modulated radiotherapy (IMRT) and image-guided radiotherapy. Management of intra- and inter-fractional prostate motion is important for the reduction of fractions. Magnetic resonance (MR)-guided online adaptive radiation therapy (MRgOART) allows the target and organ contours to be modified, and the treatment plan adapted for each session is based on the location of the organs of patients, as revealed by daily MRI. In addition, real-time monitoring of organ motion using cine-MRI can immediately recognize any dislocation during irradiation [1]. However, adapting the contours and treatment plans for each session requires significant time and effort from the medical staff [2–4]. The treatment planning system can adapt the organ contours of the planning MRI to the shape of the organs in each session via deformable image registration (DIR). DIR technology is expected not only to save time and human resources but also to standardize contours across different sessions by reducing variability, leading to a more consistent and effective treatment.

In the Elekta Unity system (Elekta AB, Stockholm, Sweden), MRgOART can be implemented using two approaches: ‘Adapt to Position’ (ATP) adjusts the treatment plan after the registration correction of the patient, and ‘Adapt to Shape’ (ATS) adjusts the treatment plan after the treatment plan and the organ contours modification to accommodate changes in organ shape [5].

ATS leverages the DIR technique to align the contours from reference MRI scans with those of daily MRI for each session [5]. Consequently, the ability to create daily treatment plans without altering the contours generated by DIR has been increasingly explored [6, 7]. Sritharan *et al.* demonstrated that treatment plans from the planning target volume (PTV) were created from contours of the prostate and seminal vesicles generated by DIR, and approximately 80% of the plans were within dose constraints, even when assessed against accurate contours [6]. Furthermore, they reported that organs at risk (OARs) met mandatory dose constraints. However, they used OAR contours that were drawn by the physician during the treatment session. Therefore, to the best of our knowledge, no reports have evaluated the feasibility of using DIR-created contours for both targets and OARs. This study aimed to assess the feasibility of treatment plans for prostate cancer generated using targets and OARs delineated by DIR, through MRgOART, which were defined DIR-plans. The feasibility of the DIR-plan was assessed using dose volume histogram (DVH) indices with clinician-created contours, specifically focusing on verifying whether the DVH indices met the dose constraints.

## MATERIALS AND METHODS

### Patient characteristics

This retrospective study was approved by the Medical Ethics Committee of our institution (HK202304–03). Due to the retrospective nature of the present study, the necessity for written informed consent was waived and an opt-out form was accessible on the website for those opting against participation. Between February 2022 and March 2023, 48 patients underwent stereotactic body radiotherapy (SBRT) with MR-Linac for prostate cancer, following hydrogel spacer implantation at our institution. Eligible patients required that the clinical target volume (CTV) consisted of the prostate and the proximal 1 cm of the seminal vesicles, with a PTV expanded 5 mm around the CTV in all directions and the prescribed dose was 37.5 Gy in five fractions. Of total, 30 out of 48 patients were eligible in this study and 150 fractions of them were evaluated.

### Treatment plan

All patients underwent planning CT and MRI at least one week after the implantation of the hydrogel spacer. Planning CT was acquired on the Aquilion Exceed LB (Canon Medical Systems, Otawara, Japan), and T2-weighted imaging was performed using an Elekta Unity. Planning CT and MRI were performed on a filled bladder and an empty rectum. The bladder wall (4 mm), rectum wall (4 mm), small bowel and sigmoid were considered the OARs. The CTV consisted of the prostate and the base of the seminal vesicles (proximal 1 cm). A uniform 5 mm expansion from the CTV was applied to create the PTV margin. The PTV for optimization (PTVopt) was created by excluding the bladder, rectum, sigmoid +5 mm and small bowel +5 mm from the PTV. The dose constraints shown in Table 1 were based on the findings of previous clinical trials and studies [8, 9]. Details regarding the workflow of MRgOART have been reported in prior publications [2, 10].

**Table 1:** Dose constraints

Structure	Dose constraint
PTVopt	D_95%_ = 37.5 Gy (prescribed dose)
	D_98%_ ≥ 36.0 Gy
	D_1 cc_ ≤ 39.4 Gy (105% prescribed dose)
Bladder wall	D_max_ ≤ 39.4 Gy (105% prescribed dose)
	D_1 cc_ ≤ 42.0 Gy
	D_53%_ ≤ 24.0 Gy
Rectum wall	D_max_ ≤ 38.6 Gy (103% prescribed dose)
	D_1 cc_ ≤ 38.5 Gy
	D_53%_ ≤ 24.0 Gy
Sigmoid	D_1 cc_ ≤ 30.0 Gy
	D_20 cc_ ≤ 25.0 Gy
Sigmoid PRV	D_0.03 cc_ ≤ 38.0 Gy
	D_20 cc_ ≤ 25.0 Gy
Small bowel PRV	D_2 cc_ ≤ 30.0 Gy

Abbreviations: PTVopt, PTV for optimization; PRV, planning organ at risk volume

### Evaluation of contours and treatment plans

**Fig. 1:**
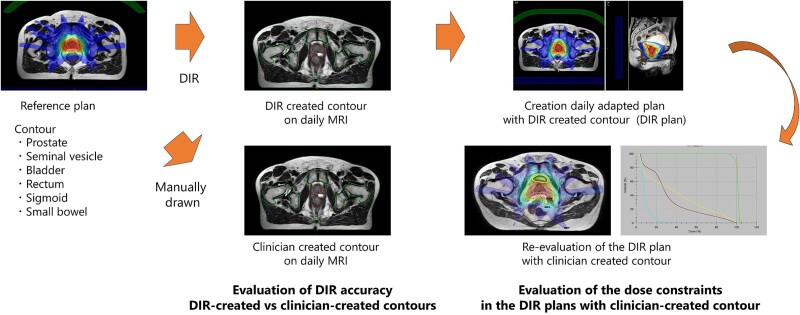
The workflow of this study. After image fusion between planning MR and daily MR, the contours of planning MR were propagated to daily MR via DIR (DIR-created contours). First, DSCs were calculated between DIR-created and clinician-created contours on daily MRI. Finally, evaluation of the dose constraints with clinician-created contour in treatment plans, which were based on the DIR-created contour to adhere to each specified dose constraint.

The workflow for evaluating the contours and treatment plans is shown in Fig. 1. The reference treatment plan was created with a dose grid of 2.5 mm, and all reference plans met all the dose constraints. Additionally, dose optimization calculations were performed to keep the minimum CTV dose as close to the prescribed dose as possible when creating the treatment plan. First, the planning MRI used in the reference plan of the patient was deformed to a daily MRI. This process propagated the contours of the prostate, seminal vesicles, bladder, rectum, sigmoid and small bowel from planning MRI to daily MRI via DIR on Monaco for Unity 5.51.11 (Elekta AB, Stockholm, Sweden), and acquired contours were defined as ‘DIR-created contours’. Radiation oncologists created contours in the planning MRI for treatment in clinical practice. Multiple radiation oncologists reviewed the contours. On daily MRI scans, a radiation oncologist manually delineated the organ contours, which were then defined as ‘Clinician-created contours’. The dice similarity coefficient (DSC) between clinician-created contours and DIR-created contours was calculated to evaluate the DIR accuracy. DSC was performed using the following formula:

A: DIR-created contours.

B: Clinician-created contours


$$ DSC=\frac{2\mid A\cap B\mid }{\left|A\right|+\mid B\mid } $$

Subsequently, a medical physicist developed treatment plans based on the DIR-created contours to adhere to each specified dose constraint. Finally, the DVH indices were evaluated using clinician-created contours. We evaluated whether all dose constraints were satisfied. At this time, only D_95%_ of the PTVopt was allowed to have a 1% deviation. In the case of distance of 5 mm or less between the sigmoid and PTV, and between the small bowel and PTV, dose constraints were evaluated for the sigmoid, sigmoid planning organ at risk volume (PRV), small bowel and small bowel PRV. Then, PRV margin was 3 mm. For patients with failed sigmoid and small bowel dose constraints, we extracted the DVH indices for each fraction and calculated the average DVH indices.

## RESULTS

The median DSC and ranges for 30 cases included 150 fractions were 0.96 (0.92–0.99) for prostate, 0.92 (0.60–0.98) for seminal vesicles, 0.97 (0.91–0.99) for CTV, 0.96 (0.92–0.98) for PTV, 0.95 (0.92–0.98) for PTVopt, 0.95 (0.31–1.00) for bladder and 0.97 (0.75–1.00) for rectum, respectively. The distance between the PTV and sigmoid was within 5 mm for 24 fractions in 10 patients, and the median DSC was 0.74 (range 0–0.94). The distance between the PTV and small bowel was within 5 mm for 10 fractions in four patients, and the median DSC was 0.54 (range 0.31–0.96).

The results of evaluating the DIR plan using clinician-created contours are revealed in Table 2. The D_95%_, D_98%_ and D_1 cc_ of the PTVopt met the dose constraints in all fractions. The D_max_ of the bladder and rectum wall exceeded the dose constraint in certain treatment fractions. However, the volumes of the exceeded dose constraints for D_max_ were within 0.03 cc. D_1 cc_ of the sigmoid and sigmoid PRV failed in only two of the 24 fractions, respectively. The two cases of failure exceeded the sigmoid dose constraint by 0.5 and 0.7%. Two cases of failure exceeded the dose constraints of the sigmoid PRV by 0.2 and 0.5%. D_2 cc_ of small bowel PRV failed in two patients. Two cases of failure exceeded the dose constraint of the small bowel PRV by 13.3 and 1%, respectively. Table 3 shows the DVH indices of all fractions for five patients whose sigmoid and small bowel dose constraints failed.

**Table 2:** The results of the evaluation of the DIR-plan using the clinician-created contour. Cases that did not meet the dose constraint values are labeled as ‘fail’, while those that met the constraints are labeled as ‘pass’

Structure	Dose constraint	Pass	Fail
PTVopt	D_95%_ = 37.5 Gy	150	0
	D_98%_ ≥ 36.0 Gy	150	0
	D_1 cc_ ≤ 39.4 Gy	150	0
Bladder wall	D_max_ ≤ 39.4 Gy	141	9
	D_0.03 cc_ ≤ 39.4 Gy	150	0
	D_1 cc_ ≤ 42.0 Gy	150	0
	D_53%_ ≤ 24.0 Gy	150	0
Rectum wall	D_max_ ≤ 38.6 Gy	140	10
	D_0.03 cc_ ≤ 38.6 Gy	150	0
	D_1 cc_ ≤ 38.5 Gy	150	0
	D_53%_ ≤ 24.0 Gy	150	0
Sigmoid	D_1 cc_ ≤ 30.0 Gy	22	2
	D_20 cc_ ≤ 25.0 Gy	24	0
Sigmoid PRV	D_0.03 cc_ ≤ 38.0 Gy	22	2
	D_20 cc_ ≤ 25.0 Gy	24	0
Small bowel PRV	D_2 cc_ ≤ 30.0 Gy	8	2

Abbreviations: PTVopt, PTV for optimization; PRV, planning organ at risk volume

**Table 3:** Detailed DVH indices of five patients whose dose constraints of sigmoid and small bowel fail. The DVH indices failed dose constraints are in bold. The last column of the table shows the average DVH indices over five fractions

	Structure	Dose constraint	First fraction	Second fraction	Third fraction	Fourth fraction	Fifth fraction	Average DVH indices
Patient 1	Sigmoid	D_1 cc_ < 30 Gy	4.1	19.4	19.2	26.0	12.3	16.2
	Sigmoid PRV	D_0.03 cc_ < 38 Gy	18.3	34.4	34.0	**38.2**	33.2	31.6
	Small bowel PRV	D_2 cc_ < 30 Gy	N/A	N/A	N/A	N/A	N/A	N/A
Patient 2	Sigmoid	D_1 cc_ < 30 Gy	16.3	25.5	3.0	28.3	26.9	20.0
	Sigmoid PRV	D_0.03 cc_ < 38 Gy	34.0	37.5	10.3	**38.1**	37.0	31.4
	Small bowel PRV	D_2 cc_ < 30 Gy	N/A	N/A	N/A	N/A	N/A	N/A
Patient 3	Sigmoid	D_1 cc_ < 30 Gy	**30.2**	28.7	15.3	27.4	23.4	25.0
	Sigmoid PRV	D_0.03 cc_ < 38 Gy	37.3	37.3	32.7	36.7	34.7	35.7
	Small bowel PRV	D_2 cc_ < 30 Gy	8.5	4.5	2.9	4.8	**34.0**	10.9
Patient 4	Sigmoid	D_1 cc_ < 30 Gy	20.0	12.3	10.9	14.7	10.7	13.7
	Sigmoid PRV	D_0.03 cc_ < 38 Gy	33.2	22.6	20.3	31.3	17.5	25.0
	Small bowel PRV	D_2 cc_ < 30 Gy	22.8	29.7	29.8	28.3	**30.3**	28.2
Patient 5	Sigmoid	D_1 cc_ < 30 Gy	24.2	25.8	28.9	**30.2**	17.8	25.4
	Sigmoid PRV	D_0.03 cc_ < 38 Gy	34.7	35.7	34.8	36.6	32.1	34.8
	Small bowel PRV	D_2 cc_ < 30 Gy	N/A	N/A	N/A	N/A	N/A	N/A

Abbreviations: PRV, planning organ at risk volume; DVH, dose volume histogram

## DISCUSSION

To the best of our knowledge, this is the first study to evaluate the DVH indices of targets and OARs with clinician-created contours in prostate MRgOART from plans developed using DIR-created contours. When the DIR-plan was evaluated using clinician-created contours, all DVH indices for the prescribed volume met the dose constraints. Regarding OAR doses, a few dose constraints in the sigmoid and small bowel failed when evaluated per fraction of the plan; however, this did not fail when assessed as an average dose over five fractions. This suggests that the DIR plan may be useful for improving the efficiency of the clinical workflow.

Furthermore, the median DSC was all high: 0.97 (0.91–0.99) for CTV, 0.96 (0.92–0.99) for prostate and 0.92 (0.6–0.98) for seminal vesicles in this study. Rasmus *et al.* reported a median DSC of 0.90 for the prostate and 0.76 for seminal vesicles in MR-MR DIR [11]. The DSC values obtained in this study were higher than those reported in a previous study. The high DSC may be a reason why many cases met the dose constraint when evaluated using clinician-created contours in this study. Previous studies have reported that it is easier to create a clinically acceptable treatment plan when the DSC of the target structure exceeds 0.95 [12]. There are two reasons for the high DSC compared with previous reports. First, all patients were implanted with hydrogel spacers. The presence of a hydrogel spacer may have increased the accuracy of DIR because the hydrogel spacer accentuated the boundaries between the prostate and rectum, and the seminal vesicles and rectum. Second, by setting the CTV up to 1 cm at the base of the seminal vesicles, we were able to reduce the influence of the variation in DIR accuracy for the seminal vesicles on the DSC of the CTV contour; thus, the DSC of the CTV and PTV showed high values.

Clinician-created contour-based dose evaluation for the DIR plan showed that the D_max_ of the bladder and rectum wall satisfied the dose constraint in more than 90% of cases. Even in cases where the dose constraint was exceeded, the deviation was within 2% of the constraint. Moreover, the constraints were met in all cases when the maximum dose was evaluated at D_0.03 cc_ for both the bladder and rectum walls. These results were obtained because the prescription volume (PTVopt) was determined by subtracting the contours of the bladder, rectum, sigmoid +5 mm and small bowel +5 mm from the PTV. As a result, there was no requirement for dose optimization calculations, which forcefully put doses into PTVs that overlapped with the bladder and rectum walls, and there were few exceedances of the dose constraints. Additionally, another factor could be that IMRT planning was designed to prevent hotspots exceeding 105% of the dose within the PTV. Furthermore, the placement of a hydrogel spacer helps reduce the dose to the OARs, which may be why few OARs exceed the dose constraints.

**Fig. 2:**
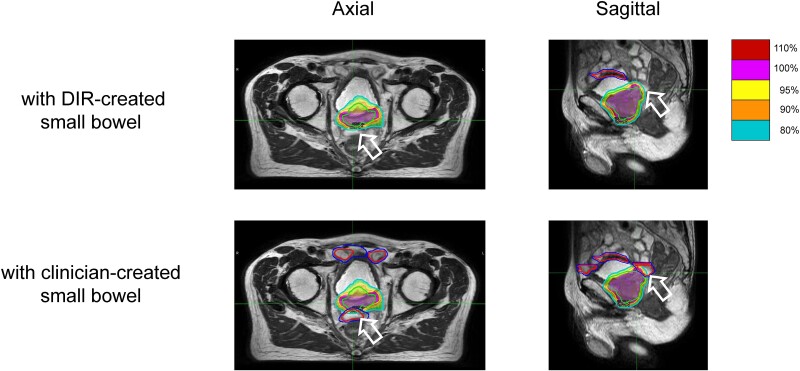
The dose distribution of the DIR plan with DIR-created (top) and clinician-created (bottom) contours. Red indicates the small bowel contours, whereas blue represents the small bowel PRV. The DIR-created small bowel contours are not adequately delineated, and when the DIR plan was re-evaluated using clinician-created small bowel contours, 30 Gy dose line irradiated the small bowel (indicated by the white arrow).

For dose constraints, there were two failures in each DVH index in the sigmoid, sigmoid PRV and small bowel PRV among the five patients. In cases where failure occurred for sigmoid and sigmoid PRV, the clinician-created contours were closer to the PTV than the DIR-created contours. Exceeded dose constraint for the small bowel was observed in cases in which the small bowel was located between the bladder and rectum on daily MRI, whereas the small bowel was located superior to the bladder on the MRI of reference plan. It was considered that the positional change in the small bowel between the planning MRI and daily MRI was too large to be handled by the DIR (Fig. 2). In this case, the D_2 cc_ of the small bowel exceeded the dose constraint by 13.3% but was within the dose constraint when evaluated using the 1–5 fraction average DVH indices (Table 3).

The results of this study indicated that a plan based on DIR-created contours can be applied to clinical cases. However, as shown in Fig. 2, dislodgements of the small bowel and sigmoid may exceed the extent of DIR propagation from the reference plan contours, and it is recommended that these contours be verified. In the present study, the treatment plan created using the DIR-created contour was clinically usable under the following conditions: hydrogel spacer was properly implanted; CTV was created with the prostate +1 cm at the base of the seminal vesicles; and the dose was prescribed to the optimized target volume such that the OARs (bladder, rectum, sigmoid and small bowel) were subtracted from the PTV. In the ATS workflow of MRgOART for patients with prostate cancer, the contouring time has been reported to exceed 10 min [13]. These results suggest that the contouring time can be significantly reduced from 10 min.

This study has a few limitations. First is the design of the PTVopt as a specific dose volume, which may not have aligned with the treatment protocols of every facility. Therefore, these findings may not be directly applicable to all facilities. This study demonstrates the potential of the DIR-plan; however, its insights highlight the need for future research to incorporate variables such as prescription methods and organ locations. These additions will refine MR-Linac workflows, aiming for efficiency and adaptability. Second, in cases where dose constraints failed in the sigmoid and small bowel, the average DVH indices over five fractions were calculated and evaluated, assuming that the hotspot (e.g. D_1 cc_ or D_2 cc_) occurred in the same location for each fraction. For a more detailed analysis, it was necessary to accumulate fractional doses using the DIR technique.

In conclusion, the present study suggested that most treatment plans created without modifying DIR-created contours (DIR-plans) were clinically viable when using a hydrogel spacer, with the CTV being the prostate plus the proximal 1 cm of the seminal vesicles and using a dose prescription volume that subtracted OARs from the PTV. However, it is recommended to verify the contours of the sigmoid and small bowel because dislodgements of the sigmoid and small bowel may exceed the extent of DIR propagation from the reference plan contours.
